# Effect of radius-dependent diffusion behavior of various gold nanoparticles on photothermal therapy

**DOI:** 10.1186/s11671-024-04031-7

**Published:** 2024-05-09

**Authors:** Donghyuk Kim, Hyunjung Kim

**Affiliations:** Department of Mechanical Engineering, Ajou University, Suwon-si, Gyeonggi-do 16499 Korea

**Keywords:** Apoptosis, Gold nanoparticles, Particle radius, Photothermal therapy, Thermal damage, Treatment starting time

## Abstract

Among the various anti-cancer treatments, photothermal therapy (PTT) is gaining traction as it is a non-invasive treatment. PTT is a treatment technique involving the use of a laser to raise the temperature of the target tumor until it dies. In this study, the effects of PTT under various conditions of squamous cell carcinoma (SCC) occurring in the skin were numerically analyzed and optimized. Gold nanoparticles (AuNPs) with different radii were injected into the center of the SCC. Subsequently, the diffusion behavior of the AuNPs was analyzed to calculate the distribution area of the AuNPs that changed over time. Furthermore, at each elapsed time point after injection, the temperature distribution in the tissue was calculated, as treatment was performed using varying laser intensities. The diffusion coefficient of AuNPs was calculated using the Stokes–Einstein equation, and diffusion behavior of AuNPs in biological tissues was analyzed using the convection–diffusion equation. Additionally, temperature distribution was analyzed using the Pennes bioheat equation. The effect of PTT under each condition was quantitatively analyzed using apoptotic variables. As a result, As the radius of AuNPs increased, the optimal treatment start time was derived as 2 h, 8 h, 8 h, and 12 h, respectively, and the laser intensity at that time was derived as 0.44 W, 0.46 W, 0.42 W, and 0.42 W, respectively. The study findings will provide reference for the optimization of the efficacy of PTT.

## Introduction

Anti-cancer treatments can be administered using various techniques [1–3]. Among them, photothermal therapy (PTT), a replacement of the existing incision treatment technique, is drawing attention as it is a non-invasive treatment [4]. PTT is a therapeutic technique based on the photothermal effect, where a targeted tumor is killed by raising its temperature [5]. Compared to existing incision treatment techniques, PTT is advantageous as it does not cause bleeding or scarring [6].

PTT involves cell death through increased temperature. Cell death occurs in various forms depending on temperature; apoptosis refers to a form of death that does not affect the surrounding tissues [7]. It occurs at 43–50 °C, and keeping the expression of apoptosis in the tumor maximized is crucial because death occurs without metastasis. In PTT, a laser is used to increase the temperature in the target area and different laser conditions are leveraged to maintain the apoptotic temperature band [8–10]. The laser wavelength is one of the various laser conditions, including irradiation time, and as the wavelength changes, the amount of laser energy absorbed by the biological tissue changes [11, 12]. If the laser is irradiated for the same amount of time, The advantage of using a wavelength band in the visible region is that the band absorbs a large amount of laser energy, allowing the medium to be heated to the target temperature even when utilizing low laser intensity. However, normal tissues, as well as the targeted tumor, can absorb laser energy, potentially causing thermal damage because of unnecessary elevations in temperature. Another disadvantage is that laser energy has a short penetration depth, resulting in treatment that can only be performed on tumors that have developed on the surface of the skin. However, using wavelengths in the near-infrared region allows for deeper penetration of the laser energy, allowing for the treatment of tumors deeper than the surface of the skin. But the tissue can only absorb a small amount of laser energy, thereby complicating elevation to the target temperature. To resolve these issues, photothermal agents (PTAs) are used in PTT to enhance light absorption at specific wavelengths [13, 14]. PTAs use localized surface plasmon resonance to enhance light absorption at specific wavelengths and have the advantage of controlling the range of temperature rise through selective injection into tumor tissues [15]. PTAs can be broadly categorized into polymer and noble metal classes based on their composition. Each class has distinct advantages and disadvantages when applied to different therapeutic situations [16, 17].

PTT has been studied in various fields, including medicine, materials science, and optics. In heat transfer studies, based on the heat transfer theory, the temperature distribution in the medium was identified through experiments and numerical analysis, and the treatment effect was indirectly confirmed. Kim et al. [18] numerically analyzed the PTT of squamous cell carcinoma (SCC) that occurred inside the skin layer. When gold nanoparticles (AuNPs) were injected into the SCC, the temperature distributions of the tumor and surrounding normal tissues were calculated by varying the laser intensity, injection time, and volume fraction of the AuNPs. Based on the calculated temperature distribution, the areas corresponding to apoptosis and necrosis were analyzed and the quantitative PTT effect in each case was derived using the apoptotic variable. These results suggest that PTT had optimal therapeutic effect. Obonai et al. [19] irradiated a biological simulant containing gold nanorods (AuNRs) and experimentally confirmed the temperature distribution in the medium under three scenarios: laser irradiation, laser irradiation with AuNRs implantation, and laser irradiation with AuNRs implantation and surface cooling. The experiments showed that when surface cooling was performed simultaneously with AuNRs implantation, higher temperatures were achieved inside the AuNRs implantation point, while the temperature of the biological simulant surface remained low. Furthermore, the measured temperature distribution was applied to the thermal damage function to determine thermal damage. Wang et al. [20] numerically analyzed the PTT of tumor tissues injected with AuNPs. The Monte Carlo method was used to calculate the laser heat distribution in the medium, and the Pennes bioheat equation was used to calculate the temperature distribution in the tumor and surrounding normal tissue located on the skin surface. Numerical analysis was performed by varying the irradiated laser intensity and volume fraction of AuNPs in each of the three scenarios. The calculated temperature distribution in each case was applied to the Arrhenius equation to determine the thermal damage to the tissue.

PTT research in the area of heat transfer is based on theoretical modeling to calculate the energy transferred within biological tissues, thereby determining the temperature distribution within the biological tissue to indirectly determine the degree of tissue death. Previous studies have mainly examined the outcomes under limited conditions and have not identified the optimal conditions for treatment. In addition, thermal damage was analyzed by focusing on the temperature at which the tumors began to die, and the degree of damage was determined based on the temperature at which the damage began to occur. However, biological tissues are subject to different forms of thermal damage depending on the temperature, and different temperature ranges cause different forms of tissue death. In addition, 43–50 °C, the temperature at which apoptosis occurs, is thermally damaging for normal tissues. However, identifying this damage is difficult because it is the target temperature for the treatment of tumor tissues. This phenomenon necessitates separate analysis of the apoptosis and necrosis temperature bands in tumor and normal tissues. Furthermore, the treatment situation was simulated by assuming that the AuNPs were distributed in a fixed form at a specific site without considering the diffusion behavior of the AuNPs. However, once injected into a biological tissue, AuNPs diffuse into the surrounding area, resulting in a nonuniform distribution of AuNPs. This diffusion behavior is influenced by the sizes of the diffusate and medium. Therefore, in this study, the diffusion behavior of AuNPs of different sizes injected into the SCC in the skin layer was analyzed, and the distribution of AuNPs according to the elapsed time after injection was determined. In addition, the effect of PTT on the SCC in the skin layer was analyzed based on the distribution of AuNPs formed through diffusion. The therapeutic effect of changing the intensity of the irradiating laser, the radius of the AuNPs, and the elapsed time after the introduction of AuNPs was determined. Finally, the effect of PTT was quantitatively analyzed using the apoptotic variable proposed by Kim et al. [21] and the conditions under which the treatment effect was maximized were determined.

## Material and methods

### Diffusion behavior of gold nanoparticles

In this study, AuNPs of different sizes were used as PTAs. The convection–diffusion equation was used to analyze the diffusion behavior of the AuNPs after injection (Eq. 1) [22].

1 $$\frac{\partial C}{{\partial \tau }} = \nabla \cdot \left( {D\nabla C} \right) - u \cdot \nabla C + R,$$

2 $$D = \frac{{K_{B} T}}{{6\pi \eta r_{np} }}.$$

where *D*, *R*, *C*, and *u* represent the particle diffusion coefficient, source or sink quantity, species concentration, and velocity, respectively. The diffusion coefficient of particle *D* was calculated using the Stokes–Einstein equation [23]. Equation (2) gives the diffusion coefficient for the situation, where a spherical particle is transported in a medium with viscosity, where *η*, *K*_*B*_, *r*_*np*_, and *T* are the dynamic viscosity of the medium, the Boltzmann constant, the radius of the particle, and the temperature of the medium, respectively. *η* was set to 8.9 × 10^–4^ Pa s [24], and *K*_*B*_ to 1.38 10^–23^ J/K.

### Optical properties of a medium

By analyzing the diffusion behavior after the injection of AuNPs, the distribution of AuNPs within the tumor at each elapsed time point after injection was determined. As the distribution of AuNPs in the tumor changed, the overall optical properties of the medium changed with changing concentrations of AuNPs. Accordingly, the Dombrovsky relation [25] was used to calculate the optical properties of the medium into which AuNPs were injected.

3 $$\mu_{abs,np} = 0.75\,f_{v} \frac{{Q_{abs,np} }}{{r_{np} }}, \quad \mu_{sca,np} = 0.75f_{v} \frac{{Q_{sca,np} }}{{r_{np} }},$$

4 $$\mu_{sca,np}^{\prime } = \mu_{sca,np} \left( {1 - g} \right),$$

5 $$\mu_{abs} = \mu_{abs,np} + \mu_{abs,m} , \mu_{sca}^{\prime } = \mu_{sca,np}^{\prime } + \mu_{sca,m}^{\prime } .$$

Equation (3) represents the light absorption (*μ*_*abs*_) and scattering coefficient (*μ*_*sca*_) according to the volume fraction of AuNPs injected into the medium. *Q* and *f*_*v*_ are the optical efficiency and volume fraction of the nanoparticles, respectively. *f*_*v*_ can be obtained from the concentration calculated at each node, and the molar mass and density of the particles. *μ*_*sca*_ is converted to the reduced scattering coefficient (*μ’*_*sca*_) via *g*, a dimensionless number that represents the direction in which light propagates, as shown in Eq. (4). After obtaining the optical properties of the AuNPs, the total optical properties of the medium were calculated as the sum of the optical properties of the medium and the nanoparticles, as shown in Eq. (5).

### Temperature behavior in a medium

The diffusion behavior and optical properties of the AuNPs in the medium calculated in previous sections can be used to analyze the temperature behavior within laser-irradiated biological tissue. In this study, the Pennes bioheat equation was used to analyze the temperature behavior in a medium for laser irradiation of biological tissues [26]. Equation (6) represents the Pennes bioheat equation with the added heat source from the laser, where *c*_*p*_, *ρ*, and *k*_*m*_ represent the specific heat, density, and thermal conductivity coefficient, respectively.

6 $$\rho c_{p} \frac{\partial T}{{\partial \tau }} = k_{m} \nabla^{2} T + q_{b} + q_{met} + q_{l} ,$$

7 $$q_{b} = \rho_{b} \omega_{b} c_{p,b} \left( {T_{b} - T} \right)$$

8 $$q_{l} = \mu_{abs} \frac{{P_{l} }}{{\pi r_{l}^{2} }}e^{{ - \mu_{tot} z}} \cdot e^{{ - \frac{{x^{2} + y^{2} }}{{r_{l}^{2} }}}} \left( {\mu_{tot} = \mu_{abs} + \mu_{sca}^{\prime } } \right).$$

In addition, *q*_*b*_, *q*_*met*_, and *q*_*l*_ represent the heat sources from the blood flow, metabolism, and laser, respectively. *q*_*b*_ can be calculated using Eq. (7), where *ω*_*b*_ is the blood perfusion rate. For *q*_*l*_, it can be calculated from *μ*_*abs*_, light attenuation coefficient (*μ*_*tot*_), and the laser's intensity (*P*_*l*_) and radius (*r*_*l*_), as shown in Eq. (8). *μ*_*tot*_ is the sum of *μ*_*abs*_ and *μ’*_*sca*_, as calculated in Sect. 2.2. This allows for the analysis of the temperature behavior in the case of laser irradiation of biological tissues.

### Numerical model and boundary conditions

**Fig. 1:**
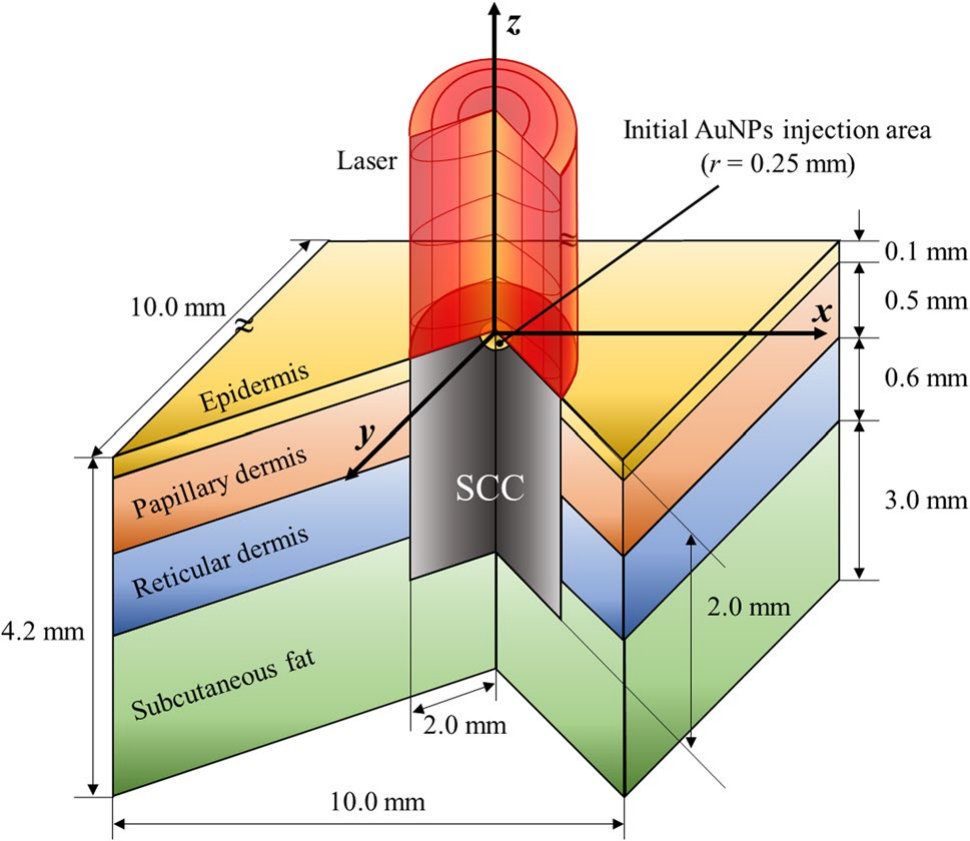
Schematic of numerical model

In this study, the diffusion behavior in tissues according to different AuNPs radii was analyzed and PTT was performed at each elapsed time point after AuNPs injection using numerical simulation modeling. Figure 1 shows a schematic of the numerical model. The skin layer consisting of the epidermis, papillary dermis, reticular dermis, and subcutaneous fat was set as a cuboid with a width of 10 mm, a length of 10 mm, and a height of 4.2 mm. Tumors that occurred in the skin layer were regarded as SCC, with a radius of 2 mm and a depth of 2 mm from the center of the skin layer. The AuNPs were distributed in a hemisphere (0.25 mm from the surface of the SCC center immediately after injection. A continuous-wave laser with a wavelength of 1064 nm and radius of 2 mm was used for treatment. The thermal and optical properties, length of each skin layer, and SCC are presented in Table 1.

**Table 1:** Various properties of skin layers and SCC [27–31]

	*t* (mm)	*k*_*m*_ (W/mK)	*ρ* (kg/m^3^)	*c*_*p*_ (J/kgK)	*ω*_*b*_ (1/s)	*μ*_*abs*_ (1/mm)	*μ′*_*sca*_ (1/mm)
Epidermis	0.1	0.235	1200	3589	0	0.4	9
Papillary dermis	0.5	0.445	1200	3300	0.0031	0.38	6
Reticular dermis	0.6	0.445	1200	3300	0.0031	0.48	2.5
Subcutaneous fat	3	0.19	1000	2674	0.0031	0.43	1
SCC	2	0.495	1070	3421	0.0063	0.047	0.221

The final goal of this study was to analyze the diffusion behavior of various *r*_*np*_ in the tissues following the injection of AuNPs and to determine the therapeutic effect of treatment at each elapsed time after injection. For the AuNPs used, a sphere type with *r*_*np*_ ranging from 10 to 40 mm was selected, and the optical efficiency of each AuNP was calculated using the discrete dipole approximation method [32, 33]. The dose of AuNPs was fixed at 300 μg/ml, and the elapsed time after injection (*τ*_*h*_) was divided into seven steps from 1 to 24 h. The treatment was assumed to have started at each *τ*_*h*_. For the laser, the irradiation time was fixed at 200 s and *P*_*l*_ was selected in 51 steps from 0 to 1 W. The conditions used in the numerical simulations are presented in Table 2.

**Table 2:** Parameters of numerical model

Parameter	Case	Number	Remarks
Radius of AuNPs (*r*_*np*_)	10 to 40 nm	4	Intv: 10 nm
Elapsed time after injection (*τ*_*h*_)	1 to 24 h	7	1, 2, 4, 8, 12, 18, 24 h
Laser power (*P*_*l*_)	0 to 1 W	51	Intv: 0.02 W

## Results and discussion

### Validation of numerical model

First, to verify its validity, the proposed numerical model was compared with that reported by Gheflati et al. [34]. The results of the experiments conducted by Paul et al. [31] were used to validate the findings of Gheflati et al. [34]. As a result, it is anticipated that a comparison and validation of the numerical modeling reported in this work with the findings of Gheflati et al. [34] research will also be able to accurately simulate the practical scenario. The validation model was set up in the same manner as that outlined by Gheflati et al. [34], with a cylindrical tumor with a radius and depth of 5 mm at the center of the normal tissue and a width, length, and height of 50, 100, and 20 mm, respectively. AuNRs were presumably injected into the center of the tumor and cylindrically distributed with a radius and height of 0.5 and 1 mm, respectively, immediately after injection. The initial injection concentration of the AuNRs was 1.661 × 10^–6^ mol/m^3^ and the external concentration was assumed to be 0 mol/m^3^. Additionally, the diffusion coefficient in the tumor was set to 9 × 10^–11^ m^2^/s and that of the normal tissue to 9 × 10^–12^ m^2^/s. The diffusion behavior was compared with the results obtained 2–4 h after injection, and the temperature change was compared with the results when laser irradiation was performed 3 h after the injection of AuNRs. The laser had intensity of 1 W/cm^2^, radius of 5 mm, and irradiation time of 300 s.

**Fig. 2:**
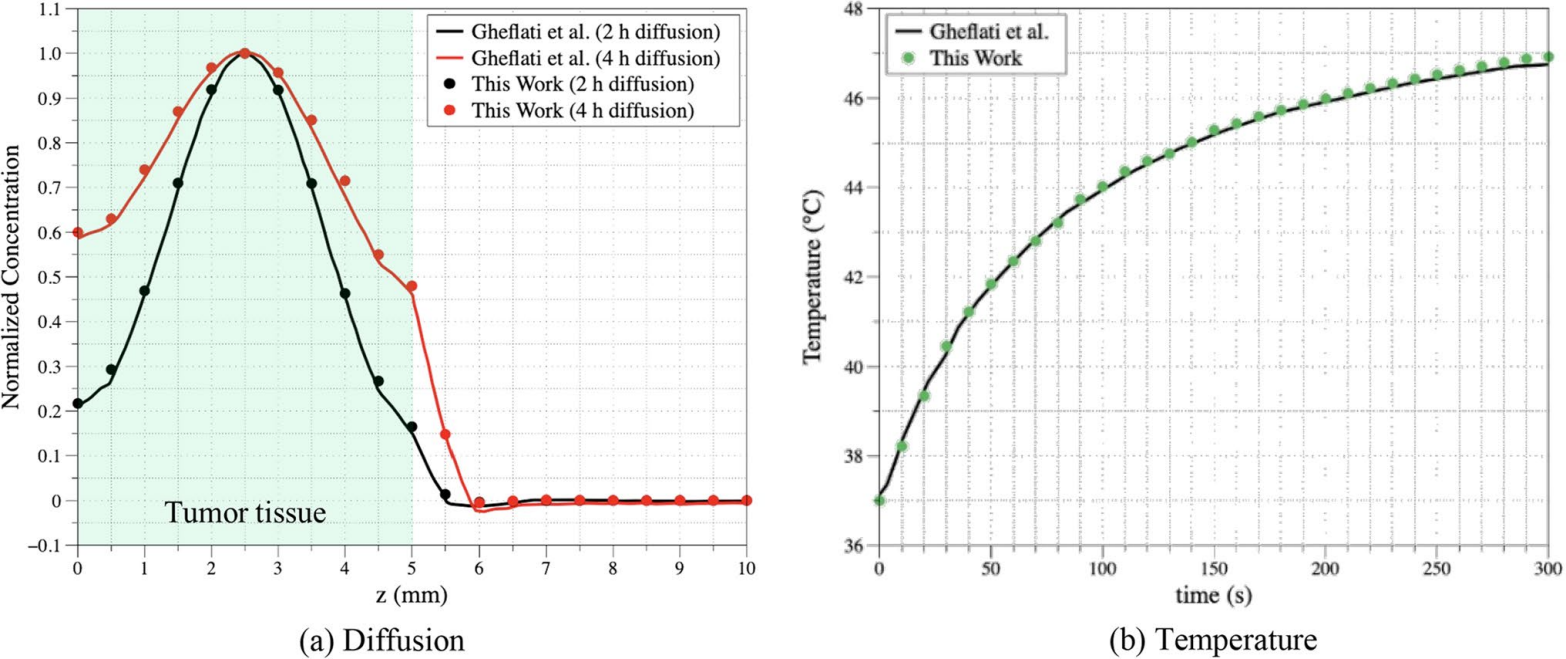
Validation results

Figure 2 shows a comparison of the diffusion behavior and temperature changes in previous studies. In the graph, the dots represent the results of the numerical model in this study, and the line represents the results obtained by Gheflati et al. [34]. Figure 2a shows an analysis of the diffusion behavior in the *z*-direction, where the green area in the graph represents the tumor region. The normalized concentration was maximum at the center of the tumor and decreased radially. Figure 2b shows the temperature change over time at the center of the tumor (*x* = 0, *z* = 2.5 mm). When comparing the results of the two graphs, the diffusion result in Fig. 2a had an average RMSE of 0.0123, and the temperature change comparison result in Fig. 2b had an RMSE of 0.1552. Thus, both the diffusion behavior and temperature change are in good agreement with those of previous studies, as seen in both graphs. This confirmed the validity of the numerical model used in this study.

### Diffusion behavior in biological tissues

When AuNPs are injected into the tumor tissue, they spread throughout the tissue over time via diffusion. As the distribution of AuNPs in tissues directly affects the degree of treatment, the diffusion behavior of AuNPs over time was analyzed in this study.

**Fig. 3:**
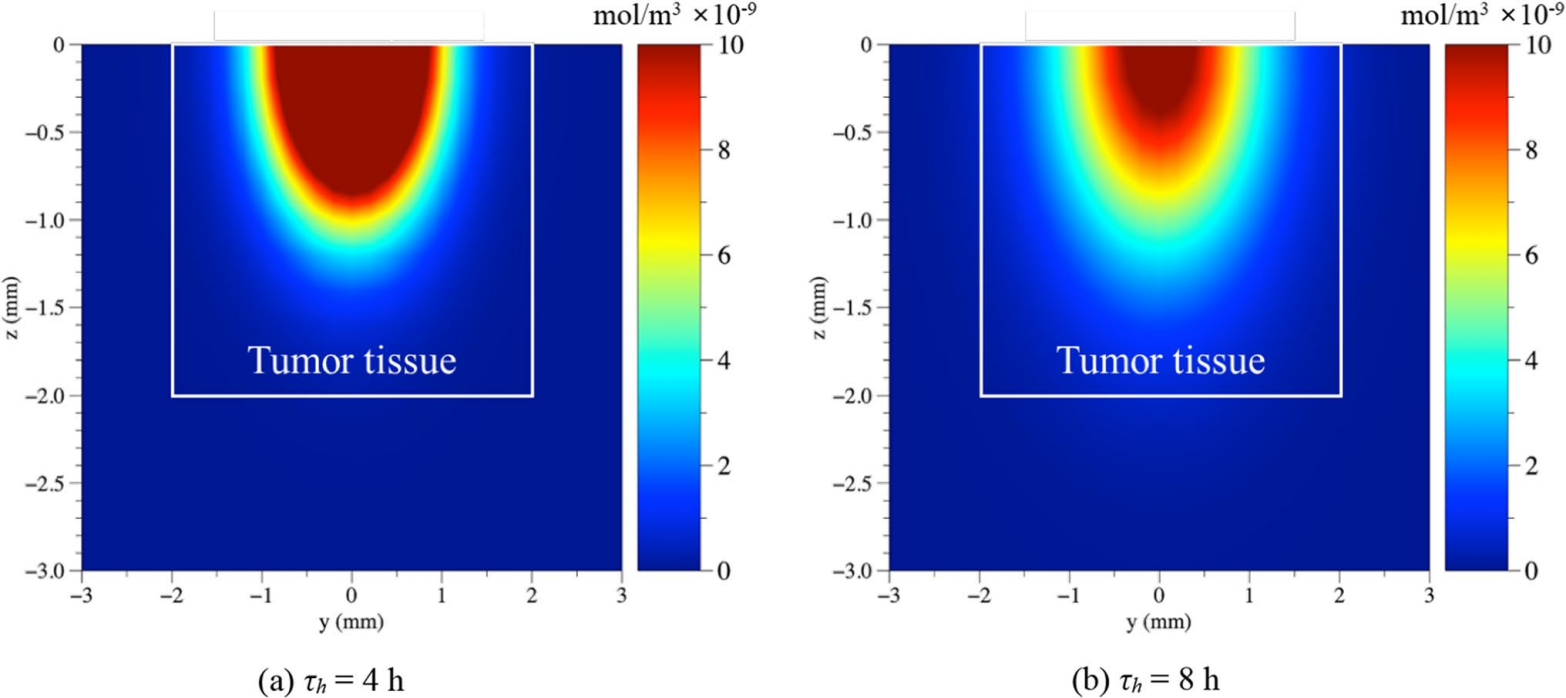
Analysis of AuNPs concentration in tissue at different *τ*_*h*_ (*r*_*np*_ = 20 nm)

Figure 3 shows the concentration of AuNPs in the tissue in the *yz* plane where *x* is zero when *τ*_*h*_ is 4 and 8 h, when AuNPs with *r*_*np*_ of 20 nm are injected into the center of the SCC. In the graph, the area inside the white box represents the SCC. Evidently, as *τ*_*h*_ increases, the distribution area of AuNPs in the tissue increases. For *τ*_*h*_ of 4 h (Fig. 3a), the distribution area of AuNPs is relatively small compared to that of *τ*_*h*_ of 8 h (Fig. 3b). This indicates that a high concentration of AuNPs was distributed within a small area because the same amount of AuNPs was injected.

**Fig. 4:**
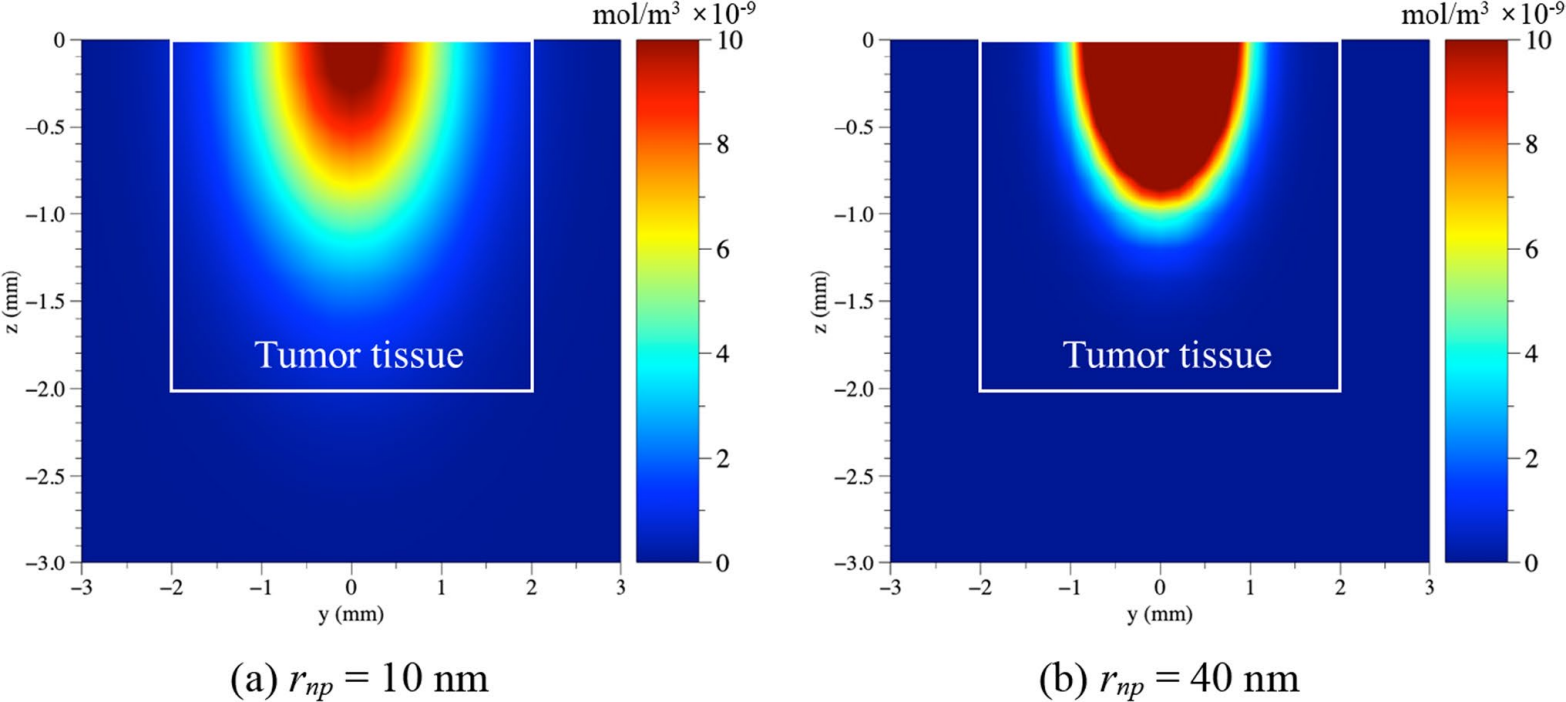
Analysis of AuNPs concentration in tissue according to different *r*_*np*_ (*τ*_*h*_ = 4 h)

Figure 4 shows the concentration of AuNPs in the tissue in the *yz* plane, where *x* is zero when *τ*_*h*_ is 4 h, when AuNPs with *r*_*np*_ of 10 and 40 nm are injected. Comparison of the case of a *r*_*np*_ of 10 nm (Fig. 4a) with that of a *r*_*np*_ of 40 nm (Fig. 4b) indicates a larger diffusion area at the same *τ*_*h*_*.* This is because the diffusion coefficient decreases with increasing *r*_*np*_, resulting in a slower diffusion rate of the AuNPs in the tissue. Furthermore, as the diffusion area becomes smaller with increasing *r*_*np*_ at the same *τ*_*h*_, the high concentration of AuNPs is distributed within a small range. Based on these considerations, the diffusion behavior under all the numerical simulation conditions selected in this study was analyzed to determine the concentration change of AuNPs with the change of *τ*_*h*_. Considering this as a basis, the temperature evolution in the tissue when the treatment is performed at each *τ*_*h*_ will be described in Sect. 3.3.

**Fig. 5:**
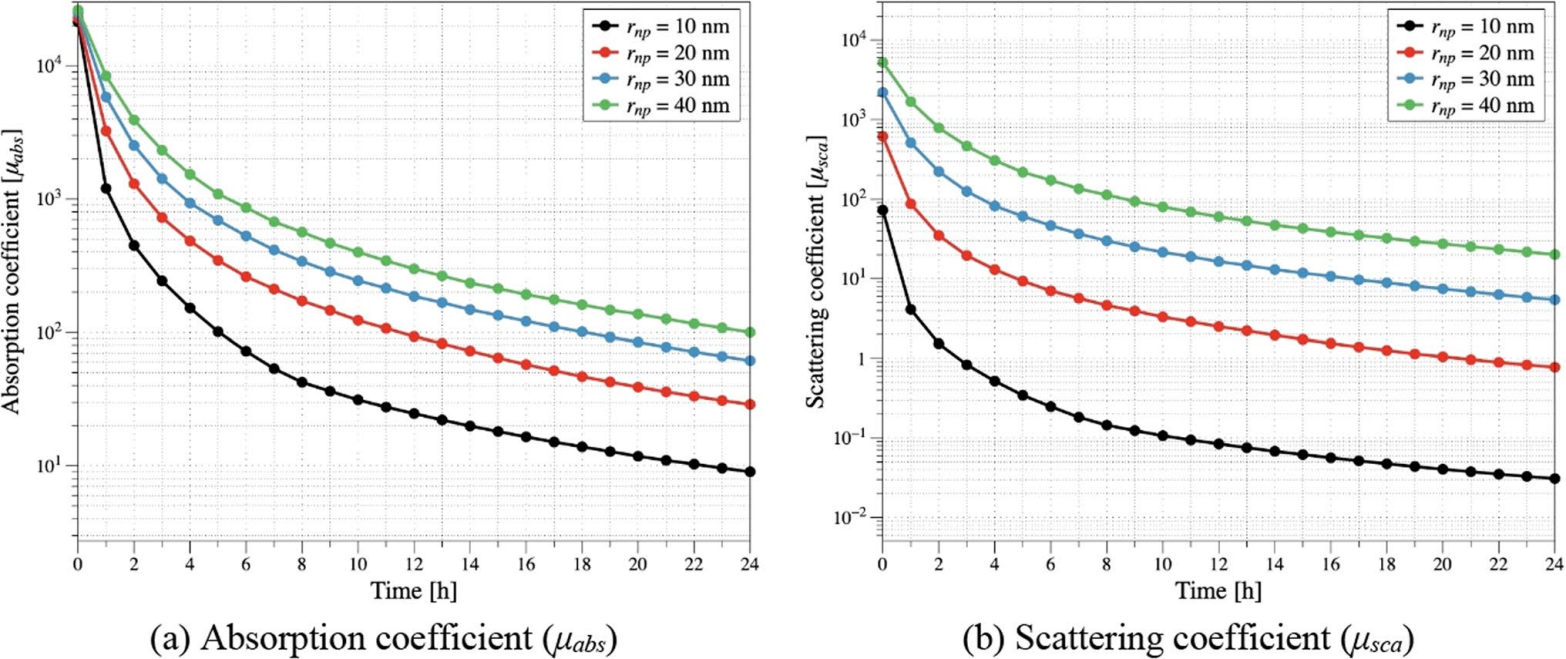
Optical coefficient for various *r*_*np*_ as a function of diffusion time (tumor center)

Figure 5 depicts the variation of the optical coefficients with respect to the diffusion time at the center of the tumor. The optical coefficients, absorption coefficient and the scattering coefficient, are calculated based on the volume fraction of the injected AuNPs, the optical efficiency, and the radius of the AuNPs, as shown in Eqs. (3) to (5). Generally, as *r*_*np*_ increases, the optical efficiency increases, leading to higher absorption and scattering coefficients at the same volume fraction. Furthermore, the calculated diffusion coefficient, calculated by Eq. (2), decreases. Therefore, at the same diffusion time, the concentration of AuNPs within the tumor is higher, resulting in higher optical coefficients. This can be observed as the slopes of the absorption and scattering coefficients decrease with increasing *r*_*np*_. Ultimately, as *r*_*np*_ increases, to maintain the apoptotic temperature, a lower laser intensity should be applied compared to when using smaller AuNPs.

### Temperature distribution in biological tissues

In the previous section, the time-dependent diffusion behavior of AuNPs in the SCC was analyzed to determine the concentration of AuNPs in the tissue at each *τ*_*h*_. Based on this information, the temperature change in tissue as the treatment was performed at each *τ*_*h*_ was determined and the region corresponding to the target temperature, the apoptosis temperature band, was analyzed.

**Fig. 6:**
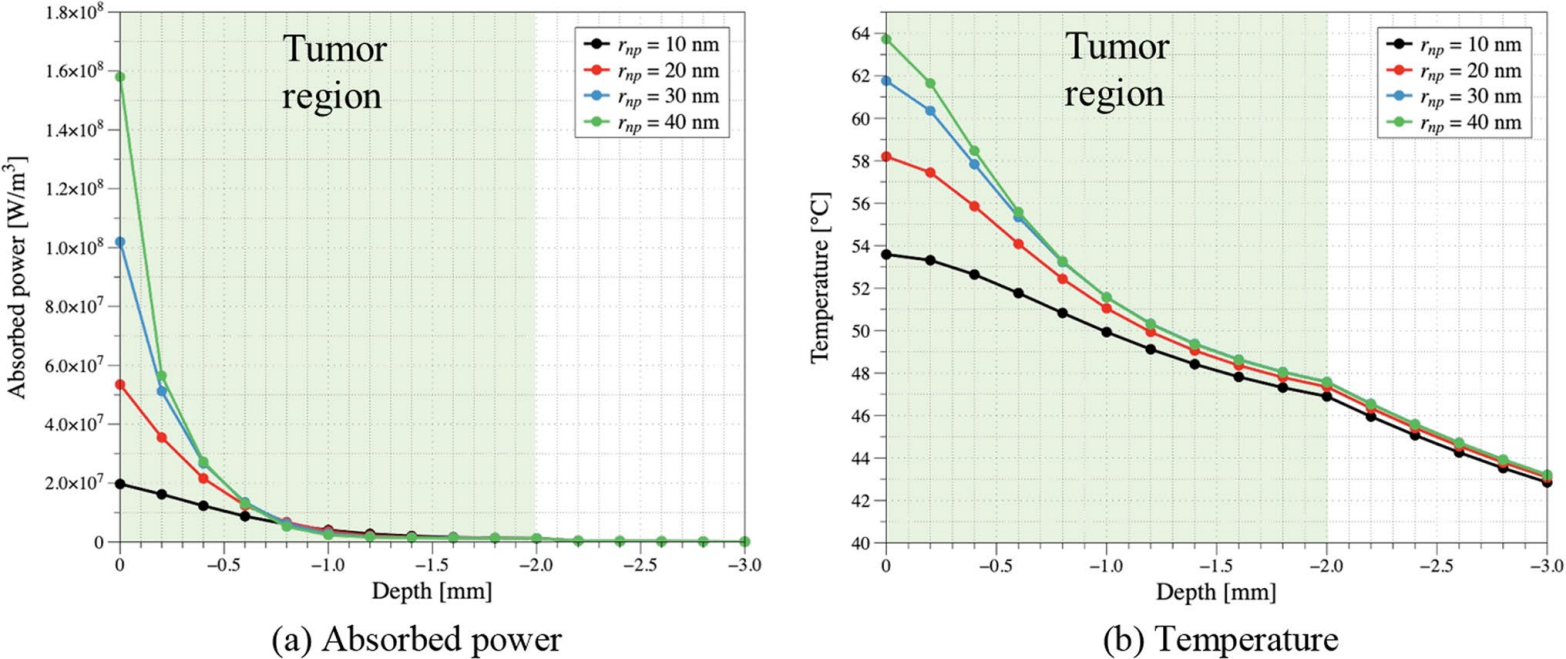
Absorbed power and temperature distribution in tissue according to *r*_*np*_ in depth direction at tumor center (*τ*_*h*_ = 2 h, *P*_*l*_ = 0.5 W, irradiated time = 200 s)

First, the absorbed power and temperature distribution by depth direction for different *r*_*np*_ were investigated. Figure 6 shows the absorbed power and temperature according to the depth direction based on the center of the tumor. The green area in the graph represents the tumor region. *τ*_*h*_ was set to 2 h, *P*_*l*_ was 0.5 W, and the laser irradiation time was 200 s. When checking the results, it can be seen that the amount of laser heat absorbed increases along the depth direction when *r*_*np*_ increases. This is the result of two factors: firstly, as the *r*_*np*_ increases, the absorption efficiency increases, leading to an increase in the calculated absorption coefficient. Secondly, as the *r*_*np*_ increases, the diffusion coefficient decreases, resulting in more AuNPs being distributed around the center within the same diffusion time. This similarly leads to an increase in the absorption coefficient, as it is equivalent to an increase in the volume fraction. Moreover, the increase in absorbed heat with the increase in *r*_*np*_ also indicates a greater rise in temperature.

**Fig. 7:**
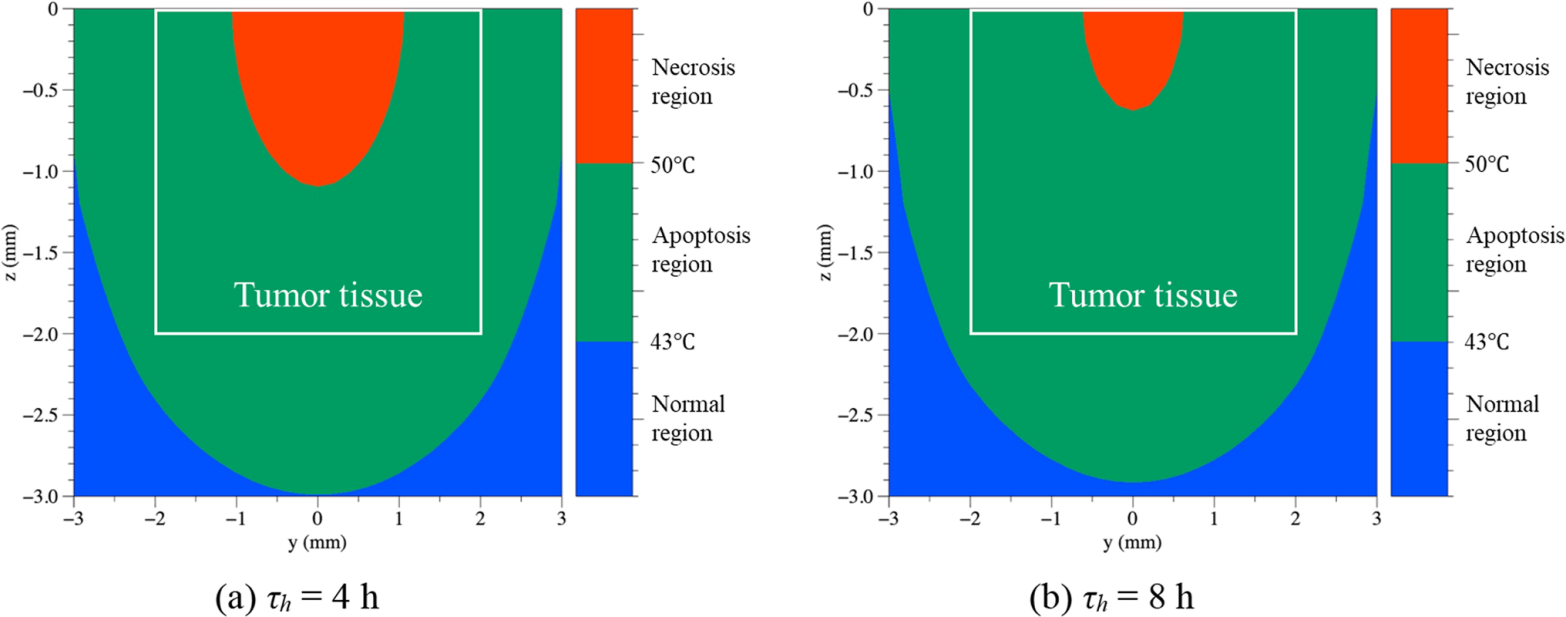
Temperature distribution in tissue at different *τ*_*h*_ (*r*_*np*_ = 20 nm, *P*_*l*_ = 0.5 W, irradiated time = 200 s)

Figure 7 shows the temperature distribution in the tissue in the *yz* plane, where *x* is zero when *τ*_*h*_ is 4 and 8 h, when *r*_*np*_ is 20 nm, *P*_*l*_ is 0.5 W, and the laser irradiation time is 200 s. In the graph, the white boxed area represents the SCC area, the red colored area represents the necrosis area above 50 °C, the green area represents the apoptosis area between 43 and 50 °C, which is the target temperature band, and the blue area represents the normal area below 43 °C. Compared to the case with *τ*_*h*_ of 4 h in Fig. 7a, the area of the apoptosis temperature band in SCC is wider in the case with *τ*_*h*_ of 8 h in Fig. 7b. The reason underlying this difference is the high concentration of AuNPs in a relatively small range in the case of *τ*_*h*_ of 4 h, as identified in Section “*Diffusion behavior in biological tissues*”, results in a higher calculated optical absorption coefficient, which leads to a greater absorption of laser energy, and consequently an excessive increase in the temperature of the medium. In Fig. 7, approximately 15% more area within the SCC fell into the apoptosis temperature band in the case with *τ*_*h*_ of 8 h than in the case with *τ*_*h*_ of 4 h.

**Fig. 8:**
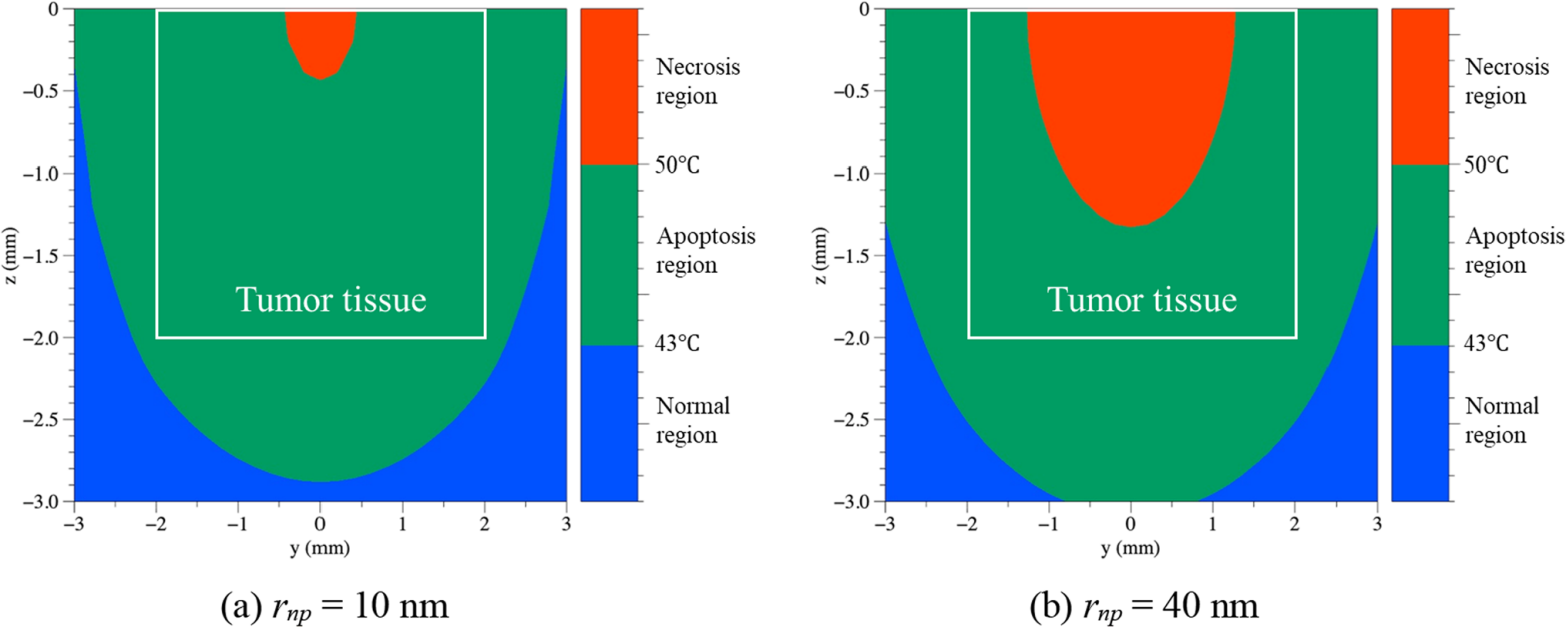
Temperature distribution in tissue according to different *r*_*np*_ (*τ*_*h*_ = 4 h, *P*_*l*_ = 0.5 W, irradiated time = 200 s)

Figure 8 shows the temperature distribution in the tissue in the *yz* plane with *x* = 0, when *r*_*np*_ is 10 and 40 nm, when *τ*_*h*_ is 4 h, *P*_*l*_ is 0.5 W, and the laser irradiation time is 200 s. For *r*_*np*_ of 10 nm (Fig. 8a), the region corresponding to the apoptosis temperature band was wider than that for *r*_*np*_ of 40 nm (Fig. 8b). As *r*_*np*_ increases, the diffusion coefficient decreases, inhibiting diffusion of the AuNPs and their distribution in high concentrations within a small area. In addition, an increase in *r*_*np*_ leads to an increase in *Q*_*a*_, which increases the calculated light-absorption coefficient of the medium. Therefore, it absorbed more laser energy, causing an excessive increase in temperature. As shown in Fig. 8, approximately 28% more necrosis occurred in the case with *r*_*np*_ of 40 nm than in the case with *r*_*np*_ of 10 nm. Accordingly, approximately 28% more areas within the SCC fell into the apoptosis temperature band in the case with *r*_*np*_ of 10 nm than in the case with *r*_*np*_ of 40 nm. Based on these considerations, the temperature distribution in the tissue was analyzed under all numerical simulation conditions presented in this study, and the PTT effects of various *τ*_*h*_, *r*_*np*_, and *P*_*l*_ values were derived from the calculated results to determine the optimal treatment conditions.

### Confirmation of apoptotic temperature retention in SCC

PTT involves irradiating the target tumor tissue with a laser and increasing its temperature. If the temperature rise is excessive, necrosis can occur, potentially leading to cancer metastasis; therefore, maintaining the apoptosis temperature band to the extent possible is important. In this study, we used the apoptotic variables proposed by Kim et al. [21]. The apoptosis retention ratio (θ^*^_A_) is a variable that quantitatively determines the degree of retention of the apoptosis temperature band in the tumor. θ^*^_A_ is defined as the average value of the ratio of the total tumor volume to the volume corresponding to the apoptosis temperature band over the total treatment time. This quantitatively elucidates the maintenance of the target temperature band and apoptotic temperature over the total treatment time.

**Fig. 9:**
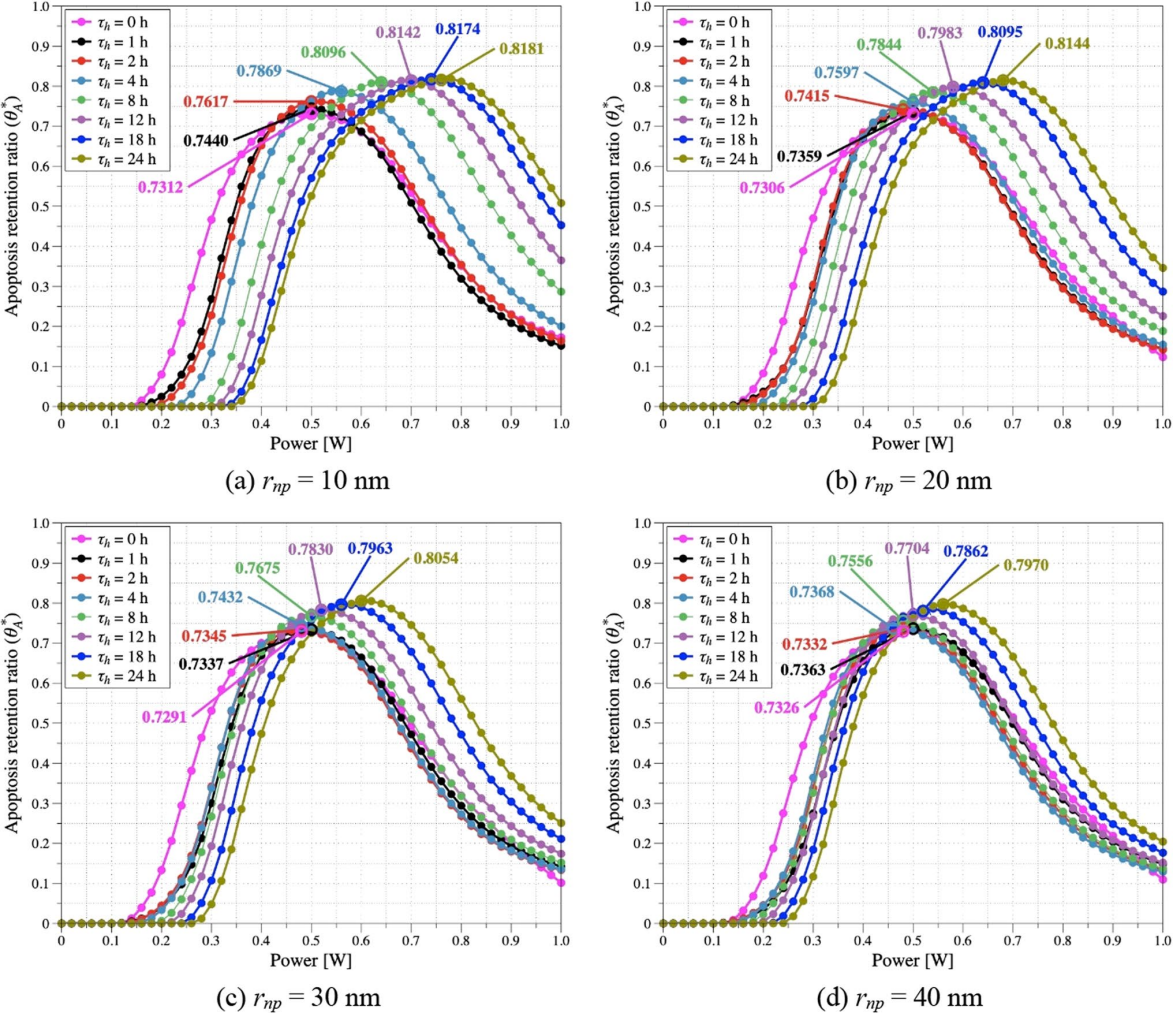
Apoptosis retention ratio (θ^*^_A_) for various *P*_*l*_ and *τ*_*h*_

Figure 9 shows θ^*^_A_ as a function of *P*_*l*_ and *τ*_*h*_ at each *r*_*np*_. Results were added when diffusion did not progress. As shown in the figure, θ^*^_A_ was found to be the minimum when no diffusion occurred in all *r*_*np*_, and the difference from the highest point was calculated to be up to 10%. Generally, θ^*^_A_ increases as *τ*_*h*_ increases for all *r*_*np*_. If *τ*_*h*_ is relatively low, the injected AuNPs cannot diffuse and are concentrated in a narrow range, resulting in a high calculated light absorption coefficient in the region. This results in excessive absorption of laser energy, causing excessive temperature rise. However, as *τ*_*h*_ increases, the distribution area of AuNPs increases and the calculated light absorption coefficient decreases, resulting in an appropriate temperature increase. This leads to an increase in the apoptotic temperature zone within the SCC, which is reflected in an increase in θ^*^_A_. Moreover, as *r*_*np*_ increases, θ^*^_A_ decreases for the same *τ*_*h*_. This is attributed to the fact that the diffusion coefficient decreases with increasing *r*_*np*_, which reduces the distribution area of AuNPs at the same *τ*_*h*_, resulting in high nonuniformity of temperature distribution. Moreover, as *r*_*np*_ increases, the deviation of the optimal *P*_*l*_ with increasing *τ*_*h*_ decreases because, as *r*_*np*_ increases, the concentrated effect of the AuNPs on the temperature increase exceeds the effect of *P*_*l*_ change.

### Confirmation of thermal damage to surrounding normal tissues

The laser energy is only absorbed by the tumor tissue, as PTT only irradiates the tumor tissue. However, the absorbed laser energy is converted to heat by the photothermal effect, causing heat transfer via conduction to the surrounding normal tissues. Accordingly, an increase in the temperature of the surrounding normal tissues occurs incidentally as treatment is performed. Analysis of the temperature of the tumor as well as surrounding normal tissue is essential because an excessive rise in temperature in the surrounding normal tissue can lead to unnecessary thermal damage. Therefore, in this study, the thermal hazard retention value (θ^*^_H_) was used to quantitatively analyze the amount of thermal damage to the surrounding normal tissue [21]. θ^*^_H_ is used to calculate the average of the weighted sum over the total treatment time, weighing each biological phenomenon differently based on the temperature. A minimum value of 1 indicates that no thermal damage occurs in the areas of the surrounding normal tissue during the total treatment time, and θ^*^_H_ increases as thermal damage occurs. This allowed for quantitative thermal damage analysis of the treatment.

**Fig. 10:**
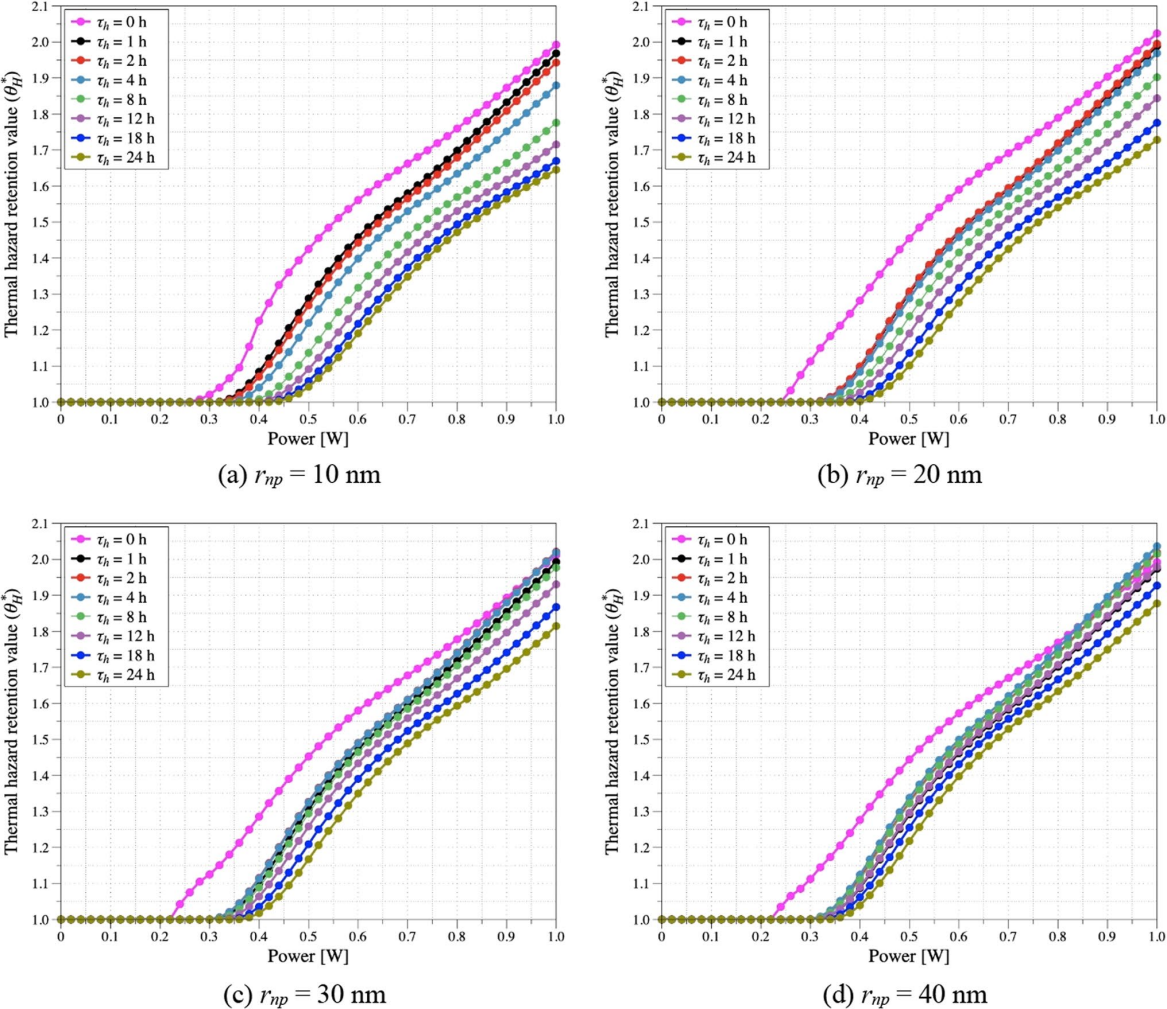
Thermal hazard retention value (θ^*^_H_) for various *P*_*l*_ and *τ*_*h*_

Figure 10 shows θ^*^_H_ as a function of *P*_*l*_ and *τ*_*h*_ at each *r*_*np*_. As same as Fig. 9, Results were added when diffusion did not progress. Overall, it was observed that θ^*^_H_ is higher when diffusion does not occur. This is attributed to the concentration of AuNPs in one location, resulting in a higher calculated absorption coefficient. Consequently, more heat is transferred to the surrounding tissue, leading to a greater increase in the temperature of the surrounding tissue. For all *r*_*np*_, θ^*^_H_ increases as *P*_*l*_ increases. This is because as *P*_*l*_ increases, applied laser energy increases which results in the increase of the amount of converted thermal energy; thereby increasing the amount of heat conducted. Furthermore, for each of the *r*_*np*_, the existence of a *τ*_*h*_ such that the thermal damage is maximized at the same *P*_*l*_ is verified. For the 10 nm *r*_*np*_ case, a lower θ^*^_H_ was obtained with increasing *τ*_*h*_ due to the fastest diffusion rate. However, for the 20, 30, and 40 nm cases of *r*_*np*_, there exists a *τ*_*h*_ such that θ^*^_H_ maximizes. As mentioned earlier, the different *r*_*np*_ changes the diffusion rate of AuNPs in the tissue, which changes the *τ*_*h*_ where the concentration of AuNPs is maximized in the inner center of the SCC. At this *τ*_*h*_, the AuNPs are evenly distributed in most areas of the SCC; therefore, the area of heat generation increases and heat transfer to the surrounding normal tissues is more efficient. As a result, θ^*^_H_ is determined to be increasing. Lastly, as *r*_*np*_ increases, θ^*^_H_ at the same *P*_*l*_ increases. This is because, as mentioned earlier, the diffusion rate of AuNPs decreases with increasing *r*_*np*_, resulting in a denser concentration of AuNPs in a narrower range at the same *τ*_*h*_, which leads to thermal conduction of the excess temperature rise near the center of the SCC to the surrounding normal tissue.

### Confirmation of quantitative treatment effect

During PTT, the temperature of the tumor and surrounding normal tissue increases simultaneously. In Sects. 3.4 and 3.5, the thermal behaviors of the tumor and surrounding normal tissue were analyzed separately, but both situations needs to be analyzed simultaneously. Therefore, in this study, the conditions for maximizing the apoptosis temperature maintenance of tumor tissue and simultaneously minimizing thermal damage to surrounding normal tissue were analyzed using the effective apoptosis retention ratio (θ^*^_eff_) [21]. θ^*^_eff_ is calculated as the ratio of θ^*^_A_ and θ^*^_H_, with a minimum value of 0 and a maximum value of 1.

**Fig. 11:**
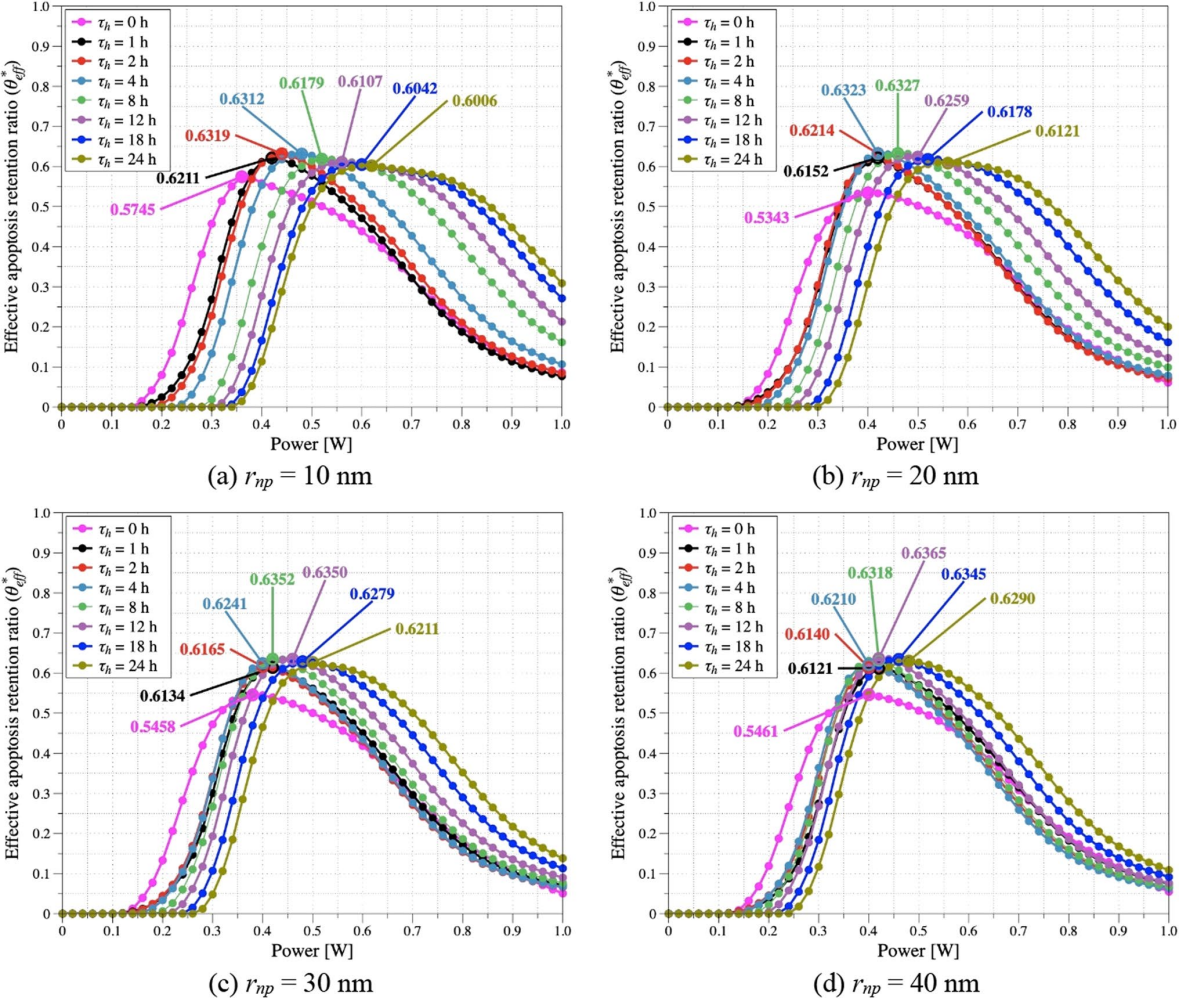
Effective apoptosis retention ratio (θ^*^_eff_) for various *P*_*l*_ and *τ*_*h*_

Figure 11 shows θ^*^_eff_ as a function of *P*_*l*_ and *τ*_*h*_ at each *r*_*np*_. When checking the graph, it was confirmed that the treatment effect was lower in all *r*_*np*_ cases when diffusion had not progressed. it was determined that there was a difference of up to 16% between maximum point. Evidently, for each *r*_*np*_, there exists a *τ*_*h*_ that produces the optimal treatment effect. The results for θ^*^_A_ show that the maximum value of θ^*^_A_ increases as *τ*_*h*_ increases, but the value of θ^*^_eff_ decreases because the value of θ^*^_H_ also increases owing to the higher *P*_*l*_ at that time. This leads to a *τ*_*h*_ that maximizes θ^*^_eff_. Furthermore, as *r*_*np*_ increases, the *τ*_*h*_ at which θ^*^_eff_ peaks increases and *P*_*l*_ decreases. An increase in *r*_*np*_ means a decrease in the diffusion rate of AuNPs, and *τ*_*h*_ must be increased to allow for a proper distribution area of AuNPs. In addition, *Q*_*a*_ increases with an increase in *r*_*np*_, which increases the light absorption coefficient; therefore, even with the same distribution area of AuNPs, a relatively low *P*_*l*_ must be applied to maintain the target temperature. Finally, the conditions under which the treatment effect was maximized for each *r*_*np*_ and the θ^*^_eff_ at the time are summarized in Table 3. Based on this information, the conditions for *τ*_*h*_ and *P*_*l*_ that optimize the effect of treatment after injection of AuNPs for various *r*_*np*_ were derived.

**Table 3:** Optimal conditions and treatment effect for various *r*_*np*_

*r*_*np*_ (nm)	*τ*_*h*_ (h)	*P*_*l*_ (W)	θ*_eff_
10	2	0.44	0.632
20	8	0.46	0.633
30	8	0.42	0.635
40	12	0.42	0.636

## Conclusions

In this study, after injecting AuNPs of various radii into the center of the SCC, the change in the distribution area over time was analyzed, and PTT at each elapsed time point was numerically analyzed. The skin layer and SCC occurring in the skin layer were modeled numerically, and the temperature change within the tissue was calculated by varying the radii of various AuNPs, the elapsed time after AuNP injection, and the intensity of the laser.

The diffusion behavior of AuNPs in biological tissues was analyzed using the convection–diffusion equation, and the temperature distribution was analyzed using the Pennes bioheat equation. The AuNPs used were spherical, and the radius of each AuNP was selected within the range of 10–40 nm. The dose of AuNPs was fixed at 300 μg/ml, and the elapsed time after injection ranged from 1 to 24 h. The wavelength of the irradiating laser was fixed at 1064 nm, and the power was varied from 0 to 1 W. The treatment was assumed to start at each elapsed time point after injection. The treatment time was fixed at 200 s and the temperature distribution within the tissue when the treatment was administered was calculated at the time elapsed after each injection.

Finally, the treatment effect in all cases was derived from the apoptotic variables, which were used to quantitatively analyze the treatment effect of PTT. The results showed that the elapsed time after injection and the laser intensity for optimal therapeutic effects varied depending on the radius of the AuNPs. As the radius of the AuNPs increased, the elapsed time after injection for optimal therapeutic effects increased, and the laser intensity decreased. Application of the optimal conditions presented in this study allowed for more accurate PTT. The results of this study were obtained by numerical analysis; therefore, clinical validation will be required in the future.

## Data Availability

Data will be made available on request.

## Abbreviations

AuNPs: Gold nanoparticles
AuNRs: Gold nanorods
PTAs: Photothermal agents
PTT: Photothermal therapy
SCC: Squamous cell carcinoma

## List of symbols

*C*: Species concentration (mol/m^3^)
*c*_*p*_: Specific heat (J/kg K)
*D*: Diffusion coefficient (m^2^/s)
*f*_*v*_: Volume fraction of AuNPs
*g*: Anisotropy factor
*K*_*B*_: Boltzmann constant (J/K)
*k*_*m*_: Thermal conductivity (W/m∙K)
*P*_*l*_: Intensity of laser (W)
*q*: Volumetric heat source (W/m^3^)
*Q*: Dimensionless efficiency factor
*R*: Source or sinks of quantity *C* (mol/m^3^)
*r*: Radius (m)
*t*: Thickness (m)
*T*: Temperature (K)
*u*: Velocity (m/s)

## Greek symbols

*η*: Dynamic viscosity (Pa s)
*θ*^***^_*A*_: Apoptosis retention ratio
*θ*^***^_*eff*_: Effective apoptosis retention ratio
*θ*^***^_*H*_: Thermal hazard retention value
*μ*: Optical coefficient (1/m)
*μ*^*′*^: Reduced optical coefficient (1/m)
*ρ*: Density ($${\text{kg}}/{{\text{m}}}^{3}$$)
*τ*: Time (s)
*τ*_*h*_: Elapsed time after injection (h)
*ω*_*b*_: Blood perfusion rate (1/s)

## Subscripts

*abs*: Absorption
*b*: Blood
*l*: Laser
*m*: Medium
*m**et*: Metabolic
*np*: Nano particle
*sca*: Scattering
*tot*: Attenuation

## References

[1] Ijaz S, Akhtar N, Khan MS, Hameed A, Irfan M, Arshad MA, Ali S, Asrar M (2018). Plant derived anticancer agents: A green approach towards skin cancers. Biomed Pharmacother.

[2] Phung DC, Nguyen HT, Tran TTP, Jin SG, Yong CS, Truong DH, Tran TH, Kim JO (2019). Combined hyperthermia and chemotherapy as a synergistic anticancer treatment. J Pharm Investig.

[3] Shen Y, Chen L, Guan X, Han X, Bo X, Li S, Sun L, Chen Y, Yue W, Xu H (2021). Tailoring chemoimmunostimulant bioscaffolds for inhibiting tumor growth and metastasis after incomplete microwave ablation. ACS Nano.

[4] Zhang L, Zhang Y, Xue Y, Wu Y, Wang Q, Xue L, Su Z, Zhang C (2019). Transforming weakness into strength: photothermal-therapy-induced inflammation enhanced cytopharmaceutical chemotherapy as a combination anticancer treatment. Adv Mater.

[5] Xu JW, Yao K, Xu ZK (2019). Nanomaterials with a photothermal effect for antibacterial activities: an overview. Nanoscale.

[6] Zou L, Wang H, He B, Zeng L, Tan T, Cao H, He X, Zhang Z, Guo S, Li Y (2016). Current approaches of photothermal therapy in treating cancer metastasis with nanotherapeutics. Theranostics.

[7] D’arcy MS (2019). Cell death: a review of the major forms of apoptosis, necrosis and autophagy. Cell Biol Int.

[8] Yang D, Yang G, Gai S, He F, An G, Dai Y, Lv R, Yang P (2015). Au 25 cluster functionalized metal–organic nanostructures for magnetically targeted photodynamic/photothermal therapy triggered by single wavelength 808 nm near-infrared light. Nanoscale.

[9] Zhen X, Pu K, Jiang X (2021). Photoacoustic imaging and photothermal therapy of semiconducting polymer nanoparticles: signal amplification and second near-infrared construction. Small.

[10] Li Y, Miao Z, Shang Z, Cai Y, Cheng J, Xu X (2020). A visible-and NIR-light responsive photothermal therapy agent by chirality-dependent MoO_3−x_ nanoparticles. Adv Func Mater.

[11] Terekhin PN, Benhayoun O, Weber ST, Ivanov DS, Garcia ME, Rethfeld B (2020). Influence of surface plasmon polaritons on laser energy absorption and structuring of surfaces. Appl Surf Sci.

[12] Guo P, Li X, Feng T, Zhang Y, Xu W (2020). Few-layer bismuthene for coexistence of harmonic and dual wavelength in a mode-locked fiber laser. ACS Appl Mater Interfaces.

[13] Liu S, Pan X, Liu H (2020). Two-dimensional nanomaterials for photothermal therapy. Angew Chem.

[14] Gao G, Sun X, Liang G (2021). Nanoagent-promoted mild-temperature photothermal therapy for cancer treatment. Adv Funct Mater.

[15] Lu M, Zhu H, Hong L, Zhao J, Masson JF, Peng W (2020). Wavelength-tunable optical fiber localized surface plasmon resonance biosensor via a diblock copolymer-templated nanorod monolayer. ACS Appl Mater Interfaces.

[16] Lv Z, He S, Wang Y, Zhu X (2021). Noble metal nanomaterials for NIR-triggered photothermal therapy in cancer. Adv Healthc Mater.

[17] Yang N, Cao C, Li H, Hong Y, Cai Y, Song X, Wang W, Mou X, Dong X (2021). Polymer-based therapeutic nanoagents for photothermal-enhanced combination cancer therapy. Small Struct.

[18] Kim D, Kim H (2023). Study on apoptosis of squamous cell carcinoma using photothermal therapy with partial injection of gold nanoparticles. Nanoscale Microscale Thermophys Eng.

[19] Obonai A, Kogawa T, Kanda Y, Oluwafemi OS, Kodama T, Komiya A (2023). Temperature distribution analysis using a combination of near-infrared laser, gold nanorods, and surface cooling equipment: temperature distribution study. Appl Therm Eng.

[20] Wang SL, Qi H, Ren YT, Chen Q, Ruan LM (2018). Optimal temperature control of tissue embedded with gold nanoparticles for enhanced thermal therapy based on two-energy equation model. J Therm Biol.

[21] Kim D, Kim H (2021). Induction of apoptotic temperature in photothermal therapy under various heating conditions in multi-layered skin structure. Int J Mol Sci.

[22] Stocker T (2011). Introduction to climate modelling.

[23] Edward JT (1970). Molecular volumes and the Stokes-Einstein equation. J Chem Educ.

[24] Soni, S, Tyagi H. Investigation of nanoparticle injection to a tissue through porous media. In: ICTEA: international conference on thermal engineering, vol 2019; 2019.

[25] Dombrovsky LA, Timchenko V, Jackson M, Yeoh GH (2011). A combined transient thermal model for laser hyperthermia of tumors with embedded gold nanoshells. Int J Heat Mass Transf.

[26] Charny CK (1992). Mathematical models of bioheat transfer. Adv Heat Transf.

[27] Çetingül MP, Herman C (2011). Quantification of the thermal signature of a melanoma lesion. Int J Therm Sci.

[28] Holmer C, Lehmann KS, Wanken J, Reissfelder C, Roggan A, Mueller G, Buhr HJ, Ritz JP (2007). Optical properties of adenocarcinoma and squamous cell carcinoma of the gastroesophageal junction. J Biomed Opt.

[29] Prasad B, Kim S, Cho W, Kim S, Kim JK (2018). Effect of tumor properties on energy absorption, temperature mapping, and thermal dose in 13.56-MHz radiofrequency hyperthermia. J Therm Biol.

[30] Salomatina E, Jiang B, Novak J, Yaroslavsky AN (2006). Optical properties of normal and cancerous human skin in the visible and near-infrared spectral range. J Biomed Opt.

[31] Paul A, Paul A (2020). Thermomechanical analysis of a triple layered skin structure in presence of nanoparticle embedding multi-level blood vessels. Int J Heat Mass Transf.

[32] Draine BT, Flatau PJ (2008). Discrete-dipole approximation for periodic targets: theory and tests. Josa a.

[33] Kim D, Kim H (2022). Numerical study on death of squamous cell carcinoma based on various shapes of gold nanoparticles using photothermal therapy. Sensors.

[34] Gheflati B, Naghavi N (2020). Computational study of nanoparticle assisted hyperthermia in tumors embedded with large blood vessels. Int J Heat Mass Transf.

